# Craniofacial features and pathogenic variants in 1,252 children with neurodevelopmental disorders

**DOI:** 10.1186/s12887-026-07267-7

**Published:** 2026-07-13

**Authors:** Ran Chen, Dandan Wu, Jerry Zhang, Yanyan Dai, Dongqing Xia, Mengying Chen, Xiaonan Li, Qianqi Liu, Yinhua Chen, Rong Li

**Affiliations:** 1 Child Healthcare Department, Children’s Hospital of Nanjing Medical University, Jiangdong South No.8 Road, Nanjing, 210008 China; 2 Child Mental Health Department, Children’s Hospital of Nanjing Medical University, Nanjing, China; 3 Basis International School Nanjing, Nanjing, Jiangsu China; 4 Nursing Department, Children’s Hospital of Nanjing Medical University, Jiangdong South No.8 Road, Nanjing, Jiangsu 210008 China

**Keywords:** Craniofacial anomalies, Neurodevelopmental disorders, Pathogenic variants

## Abstract

**Objective:**

Neurodevelopmental disorders (NDDs) are a group of highly heterogeneous diseases with complex genetic etiology. Craniofacial anomalies are frequently observed in children with NDDs. This study aimed to identify craniofacial features in children with NDDs for early warning of genetic NDDs.

**Methods:**

Peripheral blood samples were collected from children with NDDs and their parents for exome sequencing and copy number variation analysis. Typical clinical characteristics of children with NDDs, including craniofacial anomalies, limb malformations, and organ deformities, were thoroughly observed and recorded.

**Results:**

Among 1,252 children with NDDs, there were 283 cases with pathogenic variants and 219 cases of craniofacial abnormalities. The positive detection rate of pathogenic variants in the craniofacial anomaly group was significantly higher than that in the overall cohort and the cohort of non-craniofacial anomaly (*P* < 0.01). Eyes were the most common craniofacial region with anomalies in children with NDDs carrying pathogenic variants, followed by the head and oral cavity. Significant differences were observed in the rates of positive characteristics in the eight craniofacial regions between children with NDDs either carrying pathogenic variants or not. The craniofacial anomalies in ≥ 3 regions indicated a positive detection rate nearly 75% of pathogenic variants.

**Conclusion:**

Craniofacial anomalies are highly predictive of pathogenic variants in children with NDDs. Moreover, the eyes, head and oral cavity are the most common craniofacial regions with anomalies in children with NDDs carrying pathogenic variants. For a definite diagnosis, genetic testing is recommended for children with unknown causes of NDDs and anomalies in three or more craniofacial regions.

**Supplementary Information:**

The online version contains supplementary material available at https://doi.org/10.1186/s12887-026-07267-7.

## Introduction

Neurodevelopmental disorders (NDDs), a group of chronic developmental brain dysfunctions, seriously compromise the physical and mental development of children or even their lifelong health. Genetic factors play an important role in the complicated pathogenesis of NDDs in children, mainly including numerical or structural abnormalities in chromosomes, copy number variations (CNVs), mutations in a single gene or multiple genes, etc.

Global developmental delay (GDD) or intellectual disability (ID), a type of NDDs with high clinical and genetic heterogeneities, affects 1%-3% of the global population [1]. GDD/ID can be divided into the syndromic and nonsyndromic forms. Syndromic GDD/ID is a condition of additional clinical features, known comorbidities, congenital multiple malformations or special facial features other than ID, such as the Down syndrome and Rett syndrome [2]. Craniofacial anomalies are a common manifestation of syndromic GDD/ID, affecting 30%-50% of ID cases with definite genetic causes [3]. Craniofacial anomalies in GDD/ID may result from perturbation of the embryological unity or molecular and physical interplay during the fetal period when the brain and face develop robustly [4]. A unique craniofacial appearance, formed during the craniofacial development, is driven by gene-networks, and heritable, but highly variable among different populations or individuals [5]. The factors contributing to craniofacial appearance are multidimensional and complex. Ke Mao et al. [6] conducted a functional enrichment analysis of genes associated with the craniofacial region, indicating a close relationship between genetic factors for the craniofacial appearance and the development of neural crest cells (NCCs). Dysregulated NCCs resulting from abnormal gene-networks or changes in surrounding microenvironments contribute to the development of craniofacial anomalies. Craniofacial anomalies are specific, stable, and easily detectable. A unique craniofacial anomaly changes subtly with age and maintains over the life in children with GDD/ID. Genetic diseases often present initially with craniofacial anomalies, but owing to their low incidences, it is difficult for even experienced physicians to make a definite diagnosis early in infancy. NDDs can only be diagnosed until the presence of obviously delayed development of the nervous system, resulting in delayed treatment and rehabilitation. Therefore, a thorough analysis of clinical manifestations of NDDs in children is expected to provide valuable information to improve clinical management.

In the present study, we performed the exome sequencing and CNV analyses of 1,252 children with undiagnosed NDDs. Furthermore, we summarized the clinical manifestations and genetic characteristics of NDDs in children. Our findings may be used in genetic counseling, early recognition, and precise treatment of NDDs, thus preventing birth defects and improving the prognosis of NDDs.

## Methods

### Participants

Enrolled were a total of 1,252 children with undiagnosed NDDs who were admitted in the Child Healthcare Department of the Children’s Hospital of Nanjing Medical University from January 2019 to December 2022, including 879 male and 373 female participants. Children under 4 years old were assessed using the Gesell Developmental Schedules and the Griffith Mental Development Scales. If scores in two or more developmental areas (including adaptability, gross motor, fine motor, language, and personal social/behavior) were more than two standard deviations below the norm, comprehensive developmental delay was considered. For children aged 4–14 years, intellectual function was measured by the Wechsler Intelligence Scale, and all enrolled children had intellectual disabilities with an intelligence quotient (IQ) below 70. In addition, autism spectrum disorder (ASD) was diagnosed according to the standardized criteria proposed by the *American Psychiatric Association’s Diagnostic and Statistical Manual*,* Fifth Edition* (DSM-5). Exclusion criteria: (1) a definite diagnosis of genetic diseases or genetic metabolic diseases based on clinical manifestations and laboratory testing; (2) NDDs caused by perinatal ischemia, hypoxic brain injury, kernicterus, central nervous system infection, poisoning, or a history of trauma. Informed consent was obtained from the guardians of all participants.

### Data collection

A set of clinical data was recorded, including the obstetric history of the mother, perinatal conditions, birth history, feeding history, growth and developmental history, medical history and family history.

Craniofacial anomalies involving the head, skin/hair, face, eyebrows, eyes, ears, nose, and oral cavity were observed in all children with NDDs. Two pediatricians and one psychiatrist jointly evaluated the craniofacial characteristics of the children before any genetic testing was performed. Craniofacial anomalies were assessed with reference to the *Elements of Morphology: General Terms for Congenital Anomalies*.

### Genetic testing

A 2 mL peripheral blood sample was collected from each child with NDDs and their parents for exome sequencing. All genetic variants in this study were detected via next-generation sequencing (NGS). The genomic deoxyribonucleic acid (DNA) was extracted using the TIANGEN Blood Genome Magnetic Genomic DNA Kit (DP329-TA) according to the manufacturer’s instructions. The extracted DNA samples were subjected to quality control using a Qubit 2.0 fluorometer and electrophoresis with 0.8% agarose gel before library preparation. Whole exome libraries were constructed as follows. Protein-coding exome enrichment was performed using xGen Exome Research Panel v2.0 (IDT, Iowa, USA) that consists of 415,115 individually synthesized and quality-controlled probes, which targets 34 Mb protein-coding region (19,433 genes) of the human genome and covers 39 Mb of end-to-end tiled probe space. High-throughput sequencing was performed by MGI DNBSEQ-T7 sequencer, and ≥ 99% of the target regions were sequenced. Filtered clean reads were aligned to the human genome reference sequence (hg19) using the Burrows-Wheeler Aligner (http://bio-bwa.sourceforge.net/) [7, 8]. Single nucleotide variants (SNVs) and insertion-deletion mutations (INDELs) were identified using the Genome Analysis Toolkit (https://software.broadinstitute.org/gatk/) [9].

CNVs detection and annotation: CANOE, CNVnator, DeviCNV and ExomeDepth were used to detect CNVs from WES data, and all CNVs were annotated to obtain additional information about the population frequencies and possible effects. The average sequencing depth was 100×, and the minimum size threshold for included CNVs was set to 100 kb. The population frequencies for CNVs were obtained from Database of Genomic Variants (DGV).To assess the inclusion of any established dosage-sensitive genes or regions and the possible impact on gene function, each CNV was evaluated against a curated set of haploinsufficient and triplosensitive genes and genomic regions obtained from ClinGen and Database of Chromosomal Imbalance and Phenotype in Humans using Ensembl Resources (DECIPHER). Fragile X syndrome was identified using the TP-PCR combined with capillary electrophoresis. Variant annotation was performed with ANNOVAR (http://annovar.openbioinformatics.org/en/latest/) [10]. Variant sites with a minor allele frequency < 0.05 were selected from the Thousand Genomes Project (http://www.1000genomes.org), Exome Variant Server (http://evs.gs.washington.edu/EVS), and ExAC (http://exac.broadinstitute.org). Pathogenicity and conservation predictions were conducted using SIFT (http://sift.jcvi.org/) [11, 12], PolyPhen-2 (http://genetics.bwh.harvard.edu/pph2/) [13–15], MutationTaster (http://www.mutationtaster.org/) [16], and GERP (http://mendel.stanford.edu/SidowLab/downloads/gerp/index.html) [17]. Splicing site analysis was performed with SPIDEX (http://www.deepgenomics.com/spidex).

Pathogenic variants were verified by Sanger sequencing. Primer pairs were designed using Primer 3.0 (http://primer3.ut.ee/) [18–20], and PCR products were sequenced on an Applied Biosystems 3130 Genetic Analyzer. Parental pedigree sequencing was performed to analyze variant co-segregation.

Pathogenicity of variants was interpreted by molecular geneticists and clinicians according to the guidelines of the American College of Medical Genetics and Genomics (ACMG). In this study, pathogenic and likely pathogenic variants were collectively referred to as diagnostically positive. For variants of uncertain significance (VUS), they were considered positive if their pathogenicity was supported by clinical phenotype, auxiliary examinations, functional validation, and familial segregation analysis [21].

### Statistical analysis

Data recording and processing were performed using EpiData 3.0 and SPSS 22.0, respectively. Enumeration data were expressed as count and percentage, and group comparisons were analyzed by the chi-square test. *P* < 0.05 indicated a significant difference.

## Results

### Clinical characteristics of children with NDDs

Among the 1,252 children with NDDs were 879 males and 373 females. A total of 208 (16.6%) children with NDDs suffered from ASD. Pathogenic variants were detected in 283 (22.6%) children with NDDs, including 206 (16.5%) cases of single gene mutations and 77 (6.2%) cases of large-fragment deletions/duplications (Table 1). Detailed genetic findings of positive variants are provided in the supplementary table of gene results. Among the 283 children with pathogenic variants, there were 157 males and 126 females. The most frequent single gene variants were found on the X chromosome (64 cases, 31.1%), followed by those on the chromosome 2 (14 cases, 6.8%). On the X chromosome, the most frequent variants were detected in *FMR1* (13 cases), followed by *MECP2* (7 cases) and *HUWE1* (5 cases). Among the 77 children with NDDs carrying large-fragment deletions/duplications were 59 cases of large-fragment deletions, 15 cases of large-fragment duplications, and 3 cases of both. A partial deletion or duplication on chromosome 7 (20 cases, 26.0%) was the most common pathogenic variants detected in children carrying large-fragment deletions/duplications, most of which led to the phenotype of Williams-Beuren syndrome (WBS). The second most frequent variants were on chromosome 15 (11 cases, 14.3%).

**Table 1:** Clinical characteristics of children with NDDs (*n* = 1,252)

Clinical characteristics	Case number (%)
Sex	
Male	879 (70.2%)
Female	373 (29.8%)
Pathogenic/Likely Pathogenic variants	
Positive	283 (22.6%)
Negative	969 (77.4%)
Genetic causes	
Single gene mutations	206 (16.5%)
Large-fragment deletions/duplications	77 (6.2%)
External morphological features	
Positive	219 (17.5%)
Negative	1,033 (82.5%)

Among the 1,252 children with GDD/ID, craniofacial anomalies, organ deformities, and limb malformations were seen in 219, 32, and 21 cases, respectively. Detailed clinical characteristics are listed in Table 2. The contents of the scale were reported in our prior work [22, 23], which were further revised and refined in this study. Representative clinical photographs of craniofacial anomalies are listed in Table 3.

**Table 2:** Clinical phenotype of children with NDDs

Clinical phenotype		Positive characteristics
Craniofacial anomalies	Head	Macrocephaly; microcephaly; abnormality of the head.
	Skin/Hair	Abnormality of skin pigmentation; cafe-au-lait spots; skin rash; fair hair; sparse hair; low anterior hairline; capillary malformation.
	Face	Micrognathia; prognathism; retrognathia; bitemporal hollowing; long face; triangular face; flat face; facial asymmetry; long philtrum; tented philtrum; prominent forehead; broad forehead; narrow forehead; coarse facial features.
	Eyebrows	Synophrys; thick eyebrow; long eyebrows; sparse eyebrow; absent eyebrow; highly arched eyebrow.
	Eyes	Long palpebral fissure; blepharophimosis; almond-shaped palpebral fissure; telecanthus; upslanted palpebral fissure; epicanthus; downslanted palpebral fissures; strabismus; nystagmus; abnormality iris morphology; abnormal sclera morphology; periorbital fullness; deeply set eye; proptosis; ptosis; sparse eyelashes; dense eyelashes; long eyelashes.
	Ears	Anotia; cryptotia; low-set ears; macrotia; microtia; abnormal pinna morphology (abnormal helix morphology; satyr ear; protruding ear; absent antihelix; large earlobe; small earlobe); preauricular skin tag.
	Nose	Depressed nasal bridge; prominent nasal bridge; wide nasal bridge; narrow nasal ridge; narrow nasal base; wide nasal base; bulbous nose; anteverted nares; enlarged naris; narrow naris; asymmetry of the nares; cleft ala nasi; absent nasal cartilage.
	Oral cavity	Cleft palate; bifid tongue; abnormality of the gingiva; abnormality of the dentition; macroglossia; protruding tongue; abnormal tongue morphology; short hard palate; tented upper lip vermilion; thick vermilion border; thin vermilion border; cleft lip; narrow mouth; downturned corners of mouth; asymmetry of the mouth; high palate.
Limb malformations		Wide intermamillary distance; short neck; webbed neck; abnormal digit morphology.
Organ deformities		Complex congenital heart defect; abnormal digestive system morphology; abnormality of the urinary system; abnormal reproductive system morphology (e.g., hypospadias, micropenis, cryptorchidism, macroorchidism, abnormal labia minora morphology).

**Table 3:**
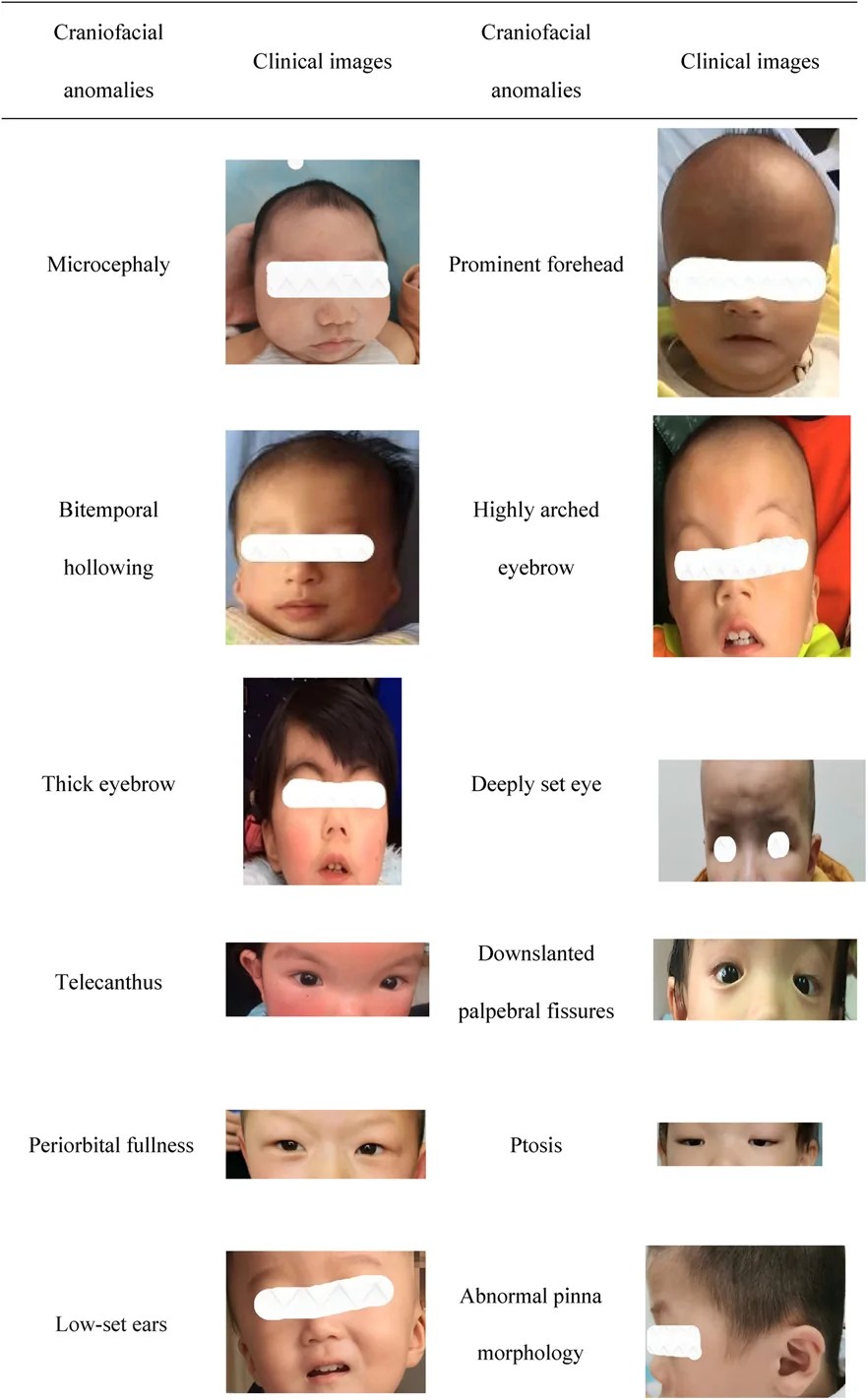

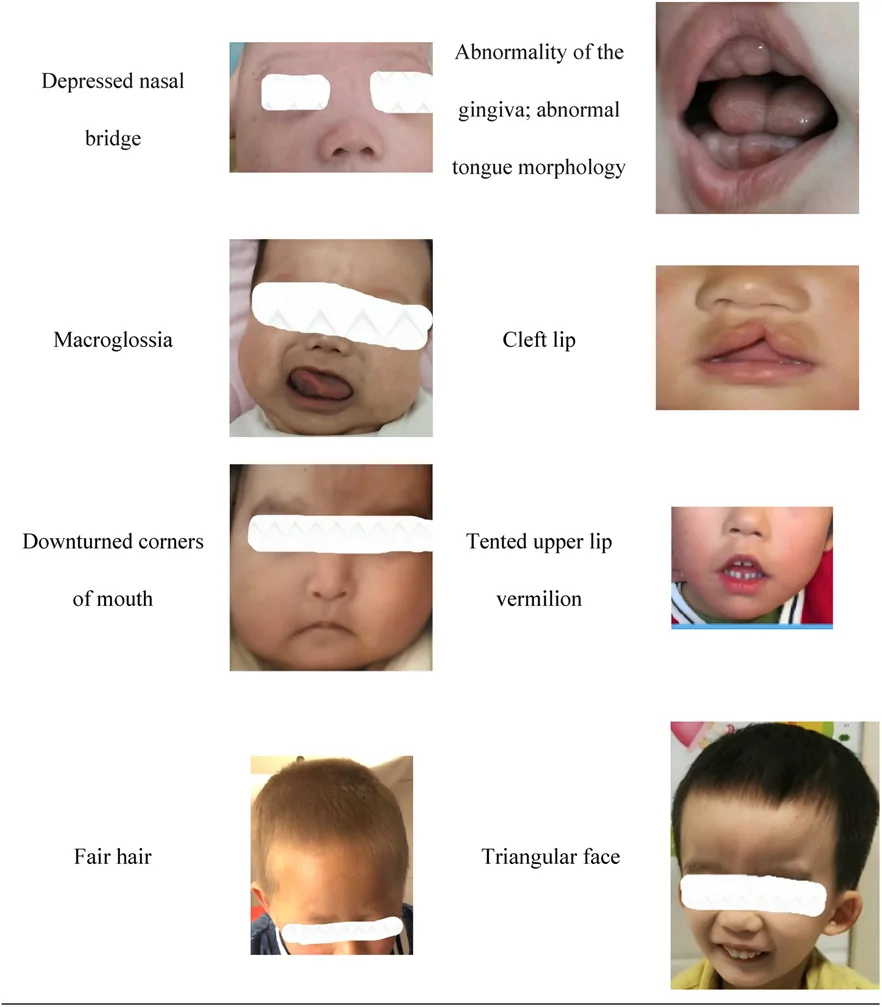
Representative clinical images of craniofacial anomalies in children with NDDs

### Clinical analysis of craniofacial anomalies in children with NDDs

The detection rate of obvious fetal malformations (e.g., congenital heart defect, limb malformations) had increased significantly with the continuous development and advancement of prenatal screening techniques. In China, induction of labor or termination of pregnancy is the most common outcome for serious fetal anomalies. As a result, organ deformities and limb malformations are rarely reported in clinical practice. In the present study, we analyzed the craniofacial anomalies in children with NDDs, which are visual features to be easily observed and recorded. Among the 1,033 (82.5%) children with NDDs who did not present craniofacial anomalies, pathogenic variants were detected in 144 (13.9%) cases. The positive detection rate of pathogenic variants in this group was significantly lower than that in the overall cohort. Among 219 (17.5%) children with NDDs presenting with craniofacial anomalies, 139 (63.5%) cases carried pathogenic variants, presenting a significantly higher positive detection rate of pathogenic variants than that in the overall cohort (Table 4).

**Table 4:** Prevalence of pathogenic variants in children with NDDs carrying craniofacial anomalies or not

	Group A vs. Group B	Group B vs. Group C	Group A vs. Group C
Pathogenic variants	22.6% (283/1,252) vs. 13.9% (144/1,033)	13.9% (144/1,033) vs. 63.5% (139/219)	22.6% (283/1,252) vs. 63.5% (139/219)
*χ*^*2*^ value	27.960	253.385	152.163
*P* value	*p* < 0.01	*p* < 0.01	*p* < 0.01

Group A includes the overall cohort of children with neurodevelopmental disorders; Group B includes those without craniofacial anomalies; Group C includes those with craniofacial anomalies

*NDDs* neurodevelopmental disorders

The proportions of children with NDDs presenting with craniofacial anomalies in each region were significantly higher in participants carrying pathogenic variants than those without detectable variants (*P* < 0.01). Multiple comparisons were corrected using the Benjamini-Hochberg false discovery rate (FDR) method. All associations remained statistically significant after FDR correction (all *P* < 0.01). Eyes were the most common craniofacial region with anomalies in children with NDDs carrying pathogenic variants, followed by the head and oral cavity (Table 5). WBS was the most common phenotype in children with NDDs carrying craniofacial anomalies, identified in 14 cases. Following WBS, pathogenic variants in *KMT2A* and *CREBBP* were the most frequent genetic causes of craniofacial anomalies, accounting for 3 cases each.

**Table 5:** Prevalence of craniofacial anomalies in each region of children with NDDs carrying pathogenic variants or not

	Pathogenic group (*n* = 283)	Non-pathogenic group (*n* = 969)	χ^2^ value/FET	*P* value	FDR-adjusted *P* value (Benjamini-Hochberg)
Eyes	69 (24.4%)	42 (4.3%)	108.947	*p* < 0.01	*p* < 0.01
Head	61 (21.6%)	29 (3.0%)	113.114	*p* < 0.01	*p* < 0.01
Oral cavity	59 (20.8%)	23 (2.4%)	122.140	*p* < 0.01	*p* < 0.01
Ears	48 (17.0%)	19 (2.0%)	97.302	*p* < 0.01	*p* < 0.01
Face	43 (15.2%)	17 (1.8%)	86.713	*p* < 0.01	*p* < 0.01
Eyebrows	32 (11.3%)	12 (1.2%)	65.489	*p* < 0.01	*p* < 0.01
Nose	29 (10.2%)	22 (2.3%)	35.668	*p* < 0.01	*p* < 0.01
Skin/hair	19 (6.7%)	3 (0.3%)	-	*p* < 0.01	*p* < 0.01

*NDDs* neurodevelopmental disorders, *FDR* false discovery rate, *FET* Fisher’s exact test

### A rating scale to detect pathogenic variants in children with NDDs carrying craniofacial anomalies

Any positive characteristic in each craniofacial region was graded as 1 point, with a total score of 0–8 points. Children with the absence of positive craniofacial characteristics in all regions were graded as 0 points. Among the 1,252 children with NDDs, there were 57, 75, 48, 24, and 15 cases graded as 1 (anomalies in one craniofacial region), 2 (anomalies in two craniofacial regions), 3 (anomalies in three craniofacial regions), 4 (anomalies in four craniofacial regions), and ≥ 5 (anomalies in at least five craniofacial regions) on the rating scale for craniofacial anomalies, respectively. Specifically, pathogenic variants were detected in 30 (52.6%), 44 (58.7%), 34 (70.8%), 18 (75%), and 13 (86.7%) cases graded as 1, 2, 3, 4, and ≥ 5 points, respectively. A significant difference in the positive detection rate of pathogenic variants was found between children with NDDs scoring 0 points and those with at least 1 point (*P* < 0.05). The positive detection rate of pathogenic variants was nearly 75% in children with NDDs carrying anomalies in three or more craniofacial regions, which was significantly higher than in those without craniofacial anomalies or with anomalies in one craniofacial region (*P* < 0.05) (Table 6).

**Table 6:** The proportion of children with NDDs graded with 0–8 points of the self-designed rating scale

	Pathogenic group (*n* = 283)	Non-pathogenic group (*n* = 969)
0 points	13.9% (144/1,033)	86.1% (889/1,033)
1 point	52.6% (30/57)^a^	47.4% (27/57)^a^
2 points	58.7% (44/75)^a^	41.3% (31/75)^a^
≥ 3points	74.7% (65/87)^ab^	25.3% (22/87)^ab^
*χ*^*2*^ value	264.487	
*P* value	*p* < 0.01	

*NDDs* neurodevelopmental disorders

a: A significant difference was obtained by the comparison to the 0-point group

b: A significant difference was obtained by the comparison to the 1-point group

## Discussion

NDDs in children are a group of heterogeneous diseases with complex pathogenic mechanisms and varying clinical manifestations. The molecular diagnosis rate of NDDs is increasing gradually with the extensive application of exome sequencing and chromosomal microarray analysis [24, 25]. Over 300 loci related to facial morphology have been identified by the genome-wide association studies, most of which are enriched in activated enhancer regions of NCCs or embryonic maxillofacial tissues [6]. Genetic variants are responsible for behavioral changes in NCCs during embryonic development, leading to a phenotypic plasticity [26]. Pathogenic variants may eventually cause neurodevelopmental disorders and malformations, including craniofacial malformations and organ deformities. Evidence has supported that genetic variants underlie the congenital craniofacial malformations [27]. The brain and the face share a common space in the developing embryo and tissues of embryonic origin. Brain morphogenesis and facial formation are usually synchronize. Besides, the pattern and pace of brain growth uniquely influence facial morphogenesis [28]. The craniofacial region is highly susceptible to malformations in genetic disorders affecting vertebrates. In this study, we also found that children with craniofacial malformations were more likely to suffer from NDDs. Craniofacial anomalies are a typical signature of early neurodevelopment [29]. Through a thorough observation, we summarized common craniofacial anomalies in children with NDDs, aiming to provide a reference for clinical management.

Exome sequencing has been recommended as a first- or second-tier test for patients with DD/ID [30]. In our study, the positive detection rate of pathogenic variants in children with NDDs carrying craniofacial anomalies increased to 63.5%, indicating that the manifestation of craniofacial anomalies is highly predictive of pathogenic variants in the cohort of NDDs. Hiraide et al. [31] reported that exome sequencing results are similar in NDD patients with either craniofacial anomalies or not. However, they suggested that the external ear malformations, such as low-set ear, macrotia, and cryptotia, may increase the detection rate of NDDs by exome sequencing. The inconsistent conclusion may be attributed to the different sample sizes and age ranges of participants. In our study, the overall cohort and the pathogenic variant-positive subgroup exhibited a skewed male-to-female ratio, consistent with the well-recognized higher prevalence of ID among males relative to females, which may also be ascribed to referral bias and gender differences in phenotypic expression or greater perinatal vulnerability among males [32]. Our analyses identified that the most frequent pathogenic single-gene variants were detected on the X chromosome. The prominent role of X-linked gene defects in ID has long been acknowledged. Furthermore, the X chromosome is highly enriched for genes critically involved in brain development and function [33]. Numerous well-established neurodevelopmental disorders are caused by pathogenic variants in X-linked genes, such as *MECP2*, *HUWE1*, *FMR1*, etc.

Craniofacial morphology is a distinctive feature of human appearance and is highly heritable. Facial formation is a process precisely regulated by genes. In recent years, many genetic loci related to craniofacial features have been discovered, and nasal traits are greatly influenced by genetic variation [5, 34, 35]. Our study inconsistently found that eyes, head, and oral cavity were the most common craniofacial regions manifesting anomalies in children with NDDs. The different conclusion could be attributed to the enrollment of a specific population of NDDs. WBS was the most common phenotype in children with NDDs carrying craniofacial anomalies, followed by Wiedemann-Steiner syndrome (caused by *KMT2A* variants) and Rubinstein-Taybi syndrome (caused by *CREBBP* variants). WBS is characterized by cardiovascular abnormalities, abnormal facial features, ID, abnormal growth, and endocrine abnormalities. Previous studies mainly focused on the cardiovascular abnormalities in WBS due to the typical structural cardiac changes of aortic stenosis [36, 37]. However, cases of WBS, recognized by developmental delay and special facial features, are relatively rare. With deeper research on WBS, a characteristic “elfin” facial gestalt has been increasingly recognized, including the facial characteristics of broad forehead, telecanthus, periorbital fullness, short nose, anteverted nares, macrotia, long philtrum, thick vermilion border, widely spaced teeth, dental malocclusion, and enamel hypoplasia. Accurate recognition of craniofacial anomalies facilitates precise genetic diagnosis.

Microcephaly was observed in many children with NDDs carrying craniofacial anomalies. Common in children with ID, microcephaly can exist alone or in combination with hereditary syndromes. Clinical manifestations of children with microcephaly vary greatly. Microcephaly can occur in children without an obvious impairment of brain function, or those with cognitive, motor, or emotional dysfunctions. Genetic variants exert a huge influence on clinical manifestations of microcephaly, which can be detectable in 15.5%-53.3% of affected children [38]. To date, many genetic variants are associated with microcephaly. These genetic alterations lead to various clinical phenotypes, resulting in a combination of specific signs and developmental anomalies, rather than isolated microcephaly. Apart from microcephaly, the most common extracranial anomalies are ocular anomalies, followed by short fingers, syndactyly, and short stature, such as the Rubinstein-Taybi syndrome, Tsukahara syndrome, and Filippi syndrome [39, 40]. A comprehensive assessment of craniofacial anomalies, especially in the head and eyes, provides useful information for the diagnosis of NDDs in children.

In-depth recognition of craniofacial anomalies has significantly increased the positive detection rate of causal genetic variants in NDDs. Three-dimensional digital imaging is a cutting-edge technique to identify facial characteristics, although it has not been broadly used in clinical practice due to the strict equipment requirements and high costs. In this study, we also found the craniofacial anomalies in ≥ 3 regions could yield a positive pathogenic variant detection rate of nearly 75%, which increased to over 85% in those with anomalies in ≥ 5 regions. Considering its high cost, genetic testing may not be accepted by all families. We therefore recommended an active genetic testing for children with unknown causes of NDDs and anomalies in three or more craniofacial regions.

### Limitations

Participants in our study were mainly from Jiangsu and Anhui Provinces, and our findings may be affected by the regional, economic, and cultural factors.

## Conclusion

Accurate recognition of craniofacial anomalies is of great significance in the genetic diagnosis of NDDs in children. It may guide testing strategies or help prioritize patients for genetic testing, providing references for a simple, effective, and accurate diagnosis of NDDs.

## Supplementary Information

Supplementary Material 1.

## Data Availability

The raw sequence data reported in this paper have been deposited in the Genome Sequence Archive (Genomics, Proteomics & Bioinformatics 2021) in National Genomics Data Center (Nucleic Acids Res 2022), China National Center for Bioinformation/Beijing Institute of Genomics, Chinese Academy of Sciences (GSA-Human: HRA003395) that are publicly accessible athttps://ngdc.cncb.ac.cn/gsa-human.

## Abbreviations

NDDs: Neurodevelopmental disorders
CNVs: Copy number variations
GDD: Global developmental delay
ID: Intellectual disability
NCCs: Neural crest cells
IQ: Intelligence quotient
ASD: Autism spectrum disorder
NGS: Next-generation sequencing
DNA: Deoxyribonucleic acid
SNVs: Single nucleotide variants
INDELs: Insertion-deletion mutations
DGV: Database of Genomic Variants
ACMG: American College of Medical Genetics and Genomics
VUS: Variants of uncertain significance
WBS: Williams-Beuren syndrome
FDR: False discovery rate
